# First-response treatment after out-of-hospital cardiac arrest: a survey of current practices across 29 countries in Europe

**DOI:** 10.1186/s13049-019-0689-0

**Published:** 2019-12-16

**Authors:** Iris Oving, Siobhan Masterson, Ingvild B.M. Tjelmeland, Martin Jonsson, Federico Semeraro, Mattias Ringh, Anatolij Truhlar, Diana Cimpoesu, Fredrik Folke, Stefanie G. Beesems, Rudolph W. Koster, Hanno L. Tan, Marieke T. Blom, for the ESCAPE-NET Investigators

**Affiliations:** 1 Department of Clinical and Experimental Cardiology, Heart Center, Amsterdam Cardiovascular Sciences, Amsterdam UMC, Department of Cardiology, Heart Center, Academic Medical Center, University of Amsterdam, Meibergdreef 9, 1105 AZ Amsterdam, The Netherlands; 2 Department of General Practice, National University of Ireland Galway and National Ambulance Service, Dublin, Ireland; 3 Norwegian National Advisory Unit on Prehospital Emergency Medicine (NAKOS), Oslo, Norway; 4 Centre for Resuscitation Science, Department for Medicine, Karolinska Institutet, Stockholm, Sweden; 5 Department of Anaesthesia, Intensive Care and Emergency Medical Services, Ospedale Maggiore, Bologna, Italy; 6 Emergency Medical Services of the Hradec Kralove Region, Czech Republic and Department of Anaesthesiology and Intensive Care Medicine, University Hospital Hradec Králové, Hradec Králové, Czech Republic; 7 Department of Emergency Medicine, “Grigore T. Popa” University of Medicine and Pharmacy, Iasi, Romania; 8 Department of Cardiology, Copenhagen University Hospital Gentofte, Hellerup, Denmark; 9 Emergency Medical Services Copenhagen, University of Copenhagen, København, Denmark; 10 Netherlands Heart Institute, Utrecht, The Netherlands

**Keywords:** First responders, Out-of-hospital cardiac arrest, Cardiopulmonary resuscitation, Europe, ESCAPE-NET

## Abstract

**Background:**

In Europe, survival rates after out-of-hospital cardiac arrest (OHCA) vary widely. Presence/absence and differences in implementation of systems dispatching First Responders (FR) in order to arrive before Emergency Medical Services (EMS) may contribute to this variation. A comprehensive overview of the different types of FR-systems used across Europe is lacking.

**Methods:**

A mixed-method survey and information retrieved from national resuscitation councils and national EMS services were used as a basis for an inventory. The survey was sent to 51 OHCA experts across 29 European countries.

**Results:**

Forty-seven (92%) OHCA experts from 29 countries responded to the survey. More than half of European countries had at least one region with a FR-system. Four categories of FR types were identified: (1) firefighters (professional/voluntary); (2) police officers; (3) citizen-responders; (4) others including off-duty EMS personnel (nurses, medical doctors), taxi drivers. Three main roles for FRs were identified: (a) complementary to EMS; (b) part of EMS; (c) instead of EMS. A wide variation in FR-systems was observed, both *between* and *within* countries.

**Conclusions:**

Policies relating to FRs are commonly implemented on a regional level, leading to a wide variation in FR-systems between and within countries. Future research should focus on identifying the FR-systems that most strongly influence survival. The large variation in local circumstances across regions suggests that it is unlikely that there will be a ‘one-size fits all’ FR-system for Europe, but examining the role of FRs in the Chain of Survival is likely to become an increasingly important aspect of OHCA research.

## Introduction

Out-of-hospital cardiac arrest (OHCA) is lethal within minutes of collapse if left untreated, and the majority of OHCA patients die before hospital admission [1, 2]. If early cardiopulmonary resuscitation (CPR) is provided, survival rate increases [3, 4]. In particular, presence of shockable rhythm is an important determinant of survival, and OHCA patients who are found with a shockable initial rhythm are more likely to survive if they are defibrillated with an automated external defibrillator (AED) [5]. However, many OHCA patients are not found in a shockable rhythm due to prolonged emergency medical services (EMS) response times, particularly in residential areas where most OHCAs occur [6–8]. When CPR is started quickly after collapse, the length of time that a shockable rhythm persists may be extended [9], thus prolonging the opportunity for successful defibrillation. Identifying and implementing systems that increase the likelihood of immediate CPR provision and rapid defibrillation are vital to improving survival. The deployment of First Responders (FRs) is one method that has been developed in order to meet this challenge.

FR-systems have been implemented differently across Europe. Some countries have expanded the traditional EMS response with dispatch of CPR trained firefighters and police officers equipped with AEDs. Research has shown that the introduction of these types of dispatched FRs led to shorter response times [10], and increased 30 day survival [11, 12]. Dispatch of trained citizen-FRs may also be successful in reducing response time [13], time to initiation of CPR [14, 15], time to defibrillation [16], and overall survival [17].

Survival rates after OHCA vary widely between regions across Europe [2] and the presence or absence of FR-systems, and differences in their implementation, may contribute to this variation. For instance, FRs may be less effective when they are inefficiently deployed and/or time from collapse to initiation of CPR is prolonged when the technology used for FR dispatch is suboptimal [15]. In addition, differences in FR skill sets may contribute, e.g., level of resuscitation training, available equipment, and experience in coping with emergency situations.

Survival rates after OHCA may increase across Europe if FR-systems are optimized. Similarly, optimization efforts may benefit from past experiences in FR implementation across Europe. However, to date, no comprehensive inventory of the different types of dispatched FR-systems used across Europe exists. Additionally, while the most recent European Resuscitation Guidelines emphasise the importance of community response in saving lives [18], the extent to which establishment of FR-systems has been adopted as national policy across Europe is unknown. Therefore, the aim of this paper is to create an inventory of dispatched FR-systems across Europe, and to determine whether countries have a national policy regarding FR-systems. This will serve as a basis to highlight key differences in order to ultimately optimise FR-systems across Europe.

## Methods

### Design and set up

This research was conducted as part of the ESCAPE-NET project that aims to discover the causes and best treatments of OHCA [19]. A mixed-method survey was combined with information retrieved from national resuscitation councils and national EMS services as a basis for an inventory.

#### Survey and information gathering

The content of the survey was determined after several meetings with an expert panel, consisting of five experts in the field of OHCA (three cardiologists, one EMS-consultant and one intensive care nurse; initials: IT, RK, AT, FS, and MR) in Europe. The survey was built by Dutch researchers and finalised after a pilot carried out by Amsterdam Resuscitation Studies (ARREST) [20] researchers.

The survey was sent to 51 OHCA professionals across 29 European countries between August and November 2018 (Additional file 1: Supplementary 1a). An OHCA professional was defined as a European Resuscitation Council (ERC) or ESCAPE-NET member with a long working experience (≥5 years) in the field of OHCA and, in particular, in prehospital resuscitation strategies. OHCA professionals were recruited during the ESCAPE-NET [19] and EuReCa [2] sessions at the ERC Congress in Bologna, 2018 (additional file 1: Supplementary 1a). Informed consent for using the contact details of the participants was sought and provided. A second attempt was made to get non-responding survey participants to take part within three weeks. All survey results were validated with the respondents before results were finalised.

In addition, data on national policies regarding FRs was sought from national resuscitation councils (or national EMS services where no council existed). If no response from a national council was received within three weeks, other national experts in the field of OHCA were consulted. Similar to the survey respondents, national experts had a long working experience (≥5 years) in the field of OHCA and were identified using the ERC or ESCAPE-NET network. Where possible, answers from survey respondents and national resuscitation councils were cross checked.

### Definitions

For the purposes of this study, EMS and FRs were defined as follows:

#### Emergency medical services (EMS)

Emergency Medical Services included on-duty emergency medical personnel who were dispatched by a dispatch centre to provide acute medical care and to transport the patient to a hospital equipped to provide acute care.

#### First responder (FR)

First Responders were defined as all individuals who were dispatched by a dispatch centre to attend OHCA events and initiate early CPR. FRs potentially included firefighters and police officers (traditional FRs) [21, 22], off-duty EMS staff and citizen-responders. An extensive description of EMS and FRs is provided in Additional file 1: Supplementary 1b.

### FR-system

The availability of dispatched FRs was determined for each country and/or region. A FR-system was defined as nationwide when it covered ≥50% of the country. The FR-system was described and characteristics were assessed (by examining each individual type of FR, as part of the FR-system). Characteristics included: recruitment and activation methods, role on scene, equipment, CPR training and frequency of training, registration, feedback, financial support, and emotional support. An extensive description of all characteristics is provided in Additional file 1: Supplementary 1c.

## Results

The response rate to the survey was 92% (47/51); information was obtained from 29 countries. For the specific questions on national policy, the response rate from national resuscitation councils was 62% (16/26; in 26 of the 29 studied countries, a national resuscitation council existed). This rate increased to 77% (20/26) after consulting other experts.

### Types and roles of FRs in Europe

**Fig. 1:**
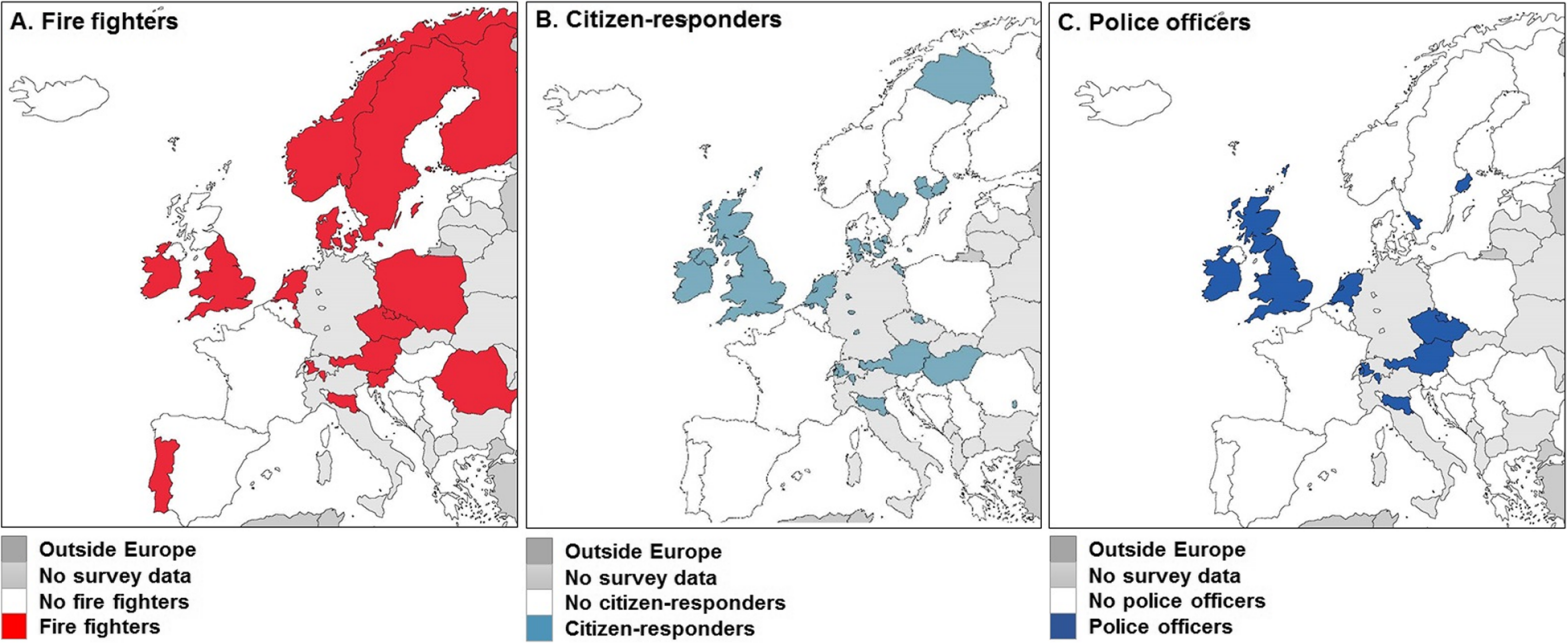
First responders per type and European region. **a**, fire fighters. **b**, citizen-responders. **c**, police officers

First responders were categorised *post-hoc* into four main types:

1. Firefighters (professional and/or voluntary) (Fig. 1a)
2. Citizen-responders (Fig. 1b)
3. Police officers (Fig. 1c)
4. Others, i.e., all responders that could not be categorized into firefighters, citizen-responders, police officers, (e.g., off-duty EMS personnel, nurses or medical doctors, and taxi drivers).

The survey identified three main roles for FRs in the event of OHCA:

1. Complementary to the statutory EMS response;
2. Part of the statutory EMS response;
3. Instead of EMS.

**Fig. 2:**
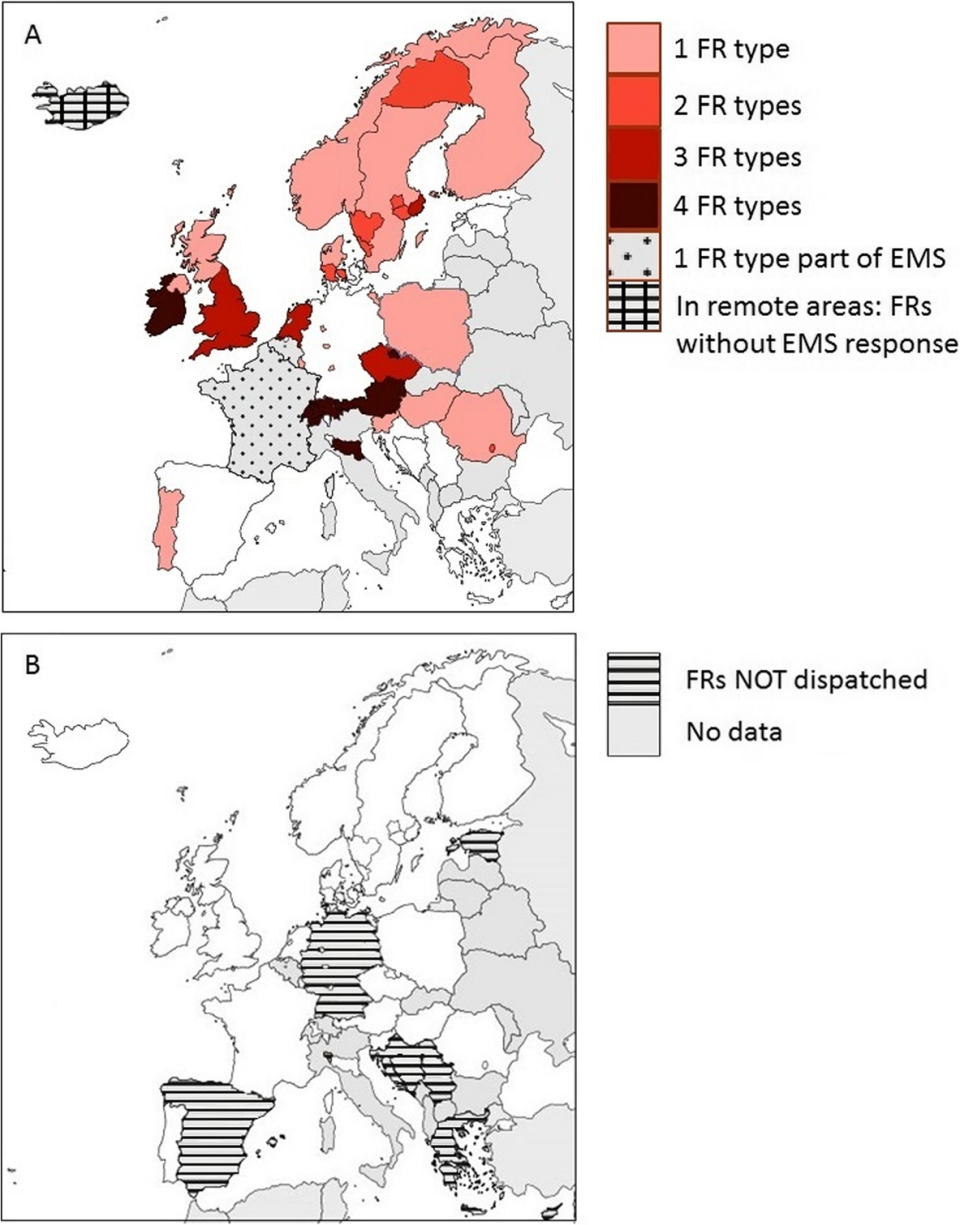
Overview of first responder systems in Europe. **a**, overview of different types of dispatched first responder systems dispatched in the event of an out-of-hospital cardiac arrest, in Europe. The number of first responder refers to the number of first responders dispatched *complementary* to the statutory Emergency Medical Services. **b**, overview of regions/countries without dispatch of first responders after an out-of-hospital cardiac arrest, in Europe. Abbreviations: FRs, First responders, EMS, Emergency Medical Services

More than half (19 of 29) of European countries or regions thereof had FR-systems (Fig. 2a). Such FR-systems were implemented nationwide in 16 countries, and regionally in 3 countries. In 14 countries, the FR-systems acted complementary to the statutory EMS response, while in one country FRs were part of the EMS response (France), and in another, FRs substituted the EMS (remote areas in Iceland). In 10 of 29 countries there was no dispatched FR-system (Fig. 2b).

### Variation in first responder-systems nationally and regionally

Variation in the type of FR-systems was observed both *between* and *within* countries. FR-systems with one FR type existed nationwide in 8 countries and regionally in 9 countries. FR-systems with two FR types existed regionally in eight countries. FR-systems including three or four FR types existed nationwide in 6 countries and regionally in 2 countries (Fig. 2a, Additional file 1: Supplementary 2).

### Characteristics of first responder-systems

Next, we analysed the characteristics of the FR types in more detail. Tables 1, 2 and 3 list the characteristics of firefighters, citizen-responders and police officers; Additional file 1: Table S1 lists the characteristics of the “other FRs”. A summarised description is provided below.

**Table 1:** Characteristics of dispatched fire fighters

Country, Region	Activation by	Location determination	1. Response needed	Role on scene	Equipment	Training; Frequency	Feedback	Financial assistance (NOT including: salary)
			2. Information provided by dispatcher					
Austria (*N* = 8.220000)	Standard communication system	GPS	1. Confirming the response 2. Location of victim; location of AED	1. Get a local AED or from the vehicle; 2. Connect a breathing mask; 3. Attach pads and follow the AED instructions; 4. Perform CPR	1. AED; 2. Safety jacket; 3. Mobile phone/pager; 4. Rescuer kit	Trained for CPR including use of an AED; Annually	Designated EMS staff discuss calls attended with firefighters	Payment of equipment; AED related disposables
Czech Republic, (*N* = 10.521646)	Standard communication system	Address and GPS	1. Subsequent call from dispatcher to firefighter or backwards 2. Location of victim; demographics; situational information	1. Get an AED from the vehicle; 2. Connect a breathing mask; 3. Attach pads and follow the AED instructions; 4. Perform CPR	1. AED; 2. Safety jacket; 3. Mobile phone/pager; 4. Rescuer kit; 5. BVM, 6. Oxygen	Trained for CPR including use of an AED; Annually	Designated EMS staff discuss calls attended with firefighters/Firefighters participate in a debrief with EMS staff immediately after an OHCA/the own training officers can also take part	No
Denmark, Capital Region, Region Zealand, Region South (*N* = 3.879024)	Standard communication system	GPS	1. No response from the firefighter is required 2. Location of victim	1. Get an AED from the vehicle; 2. Attach pads and follow the AED instructions; 3. Perform CPR	1. AED	Trained for CPR including use of an AED; Annually	None	No
Denmark Central Region, Region North (*N* = 1.903148)	Standard communication system	GPS	1. Confirming the response 2. Location of victim	1. Get an AED from the vehicle; 2. Attach pads and follow the AED instructions; 3. Perform CPR	1. AED; 2. Safety jacket; 3. Mobile phone/pager; 4. Rescuer kit	Trained for CPR including use of an AED; Introduced low dose, high frequency training	None	No
England (*N* = 55.980000)	SMS or APP	Address	1. No response from the firefighter is required 2. Location of victim, demographics (victim) & situational info	1. Get an AED from the vehicle; 2. Connect a breathing mask; 3. Attach pads and follow the AED instructions; 4. Perform CPR; 5. They are trained in airway management OP airways	1. AED; 2. Mobile phone/pager	Trained for CPR including use of an AED; Annually	Firefighter activity is described in the annual report/Firefighters receive a regular report of their activity/Firefighters receive written feedback on individual calls from the EMS	No
Finland (*N* = 5.513000)	Standard communication system	GPS	1. Confirming the response 2. Location of victim	1. Get an AED from the vehicle; 2. Connect a breathing mask; 3. Attach pads and follow the AED instructions; 4. Perform CPR	1. AED; 2. Safety jacket; 3. Mobile phone/pager; 4. Rescuer kit	Trained for CPR including use of an AED; Annually	Firefighter activity is described in the annual report	No
Ireland (*N* = 4.830000)	Standard communication system; SMS	Not determined	1. Following local fire and rescue dispatch protocols 2. Location of victim; demographics; situational information	1. Get a local AED or from the vehicle; 2. Connect a breathing mask; 3. Attach pads and follow the AED instructions; 4. Perform CPR	1. AED; 2. Safety jacket; 3. Mobile phone/pager; 4. Resuscitation kit	Trained for CPR including use of an AED; *Bi-annually*	Firefighter activity is described in the annual report/Firefighters participate in a debrief with EMS staff immediately after an OHCA	No
Italy, Emilia Romagna (*N* = 4.453000)	APP	GPS	1. Confirming the response 2. Location of victim & location of AED	1. Get a local AED or from the vehicle; 2. Connect a breathing mask; 3. Attach pads and follow the AED instructions; 4. Perform CPR	1. AED; 2. Safety jacket; 3. Mobile phone/pager; 4. Rescuer kit	Trained for CPR including use of an AED; *Bi-*annually	The responder manager sends an email to collect feedback by mail (but is not a routine)	Supported by Emilia Romagna Healthcare Region Funding and Fondazione del Monte di Bologna e Ravenna for development.
Luxembourg (*N* = 6,020,050)	Standard communication system; SMS, Pager	Not determined	1. Following local fire and rescue dispatch protocols 2. Location of victim; demographics; situational information	1. Get an AED from the vehicle; 2. Attach pads and follow the AED instructions; 3. Perform CPR	1. AED; 2. Safety jacket; 3. Mobile phone/pager; 4. Rescuer kit	Trained for CPR including use of an AED; initial advanced training in first aid (~ 40 h)*,* annually 8 h training	None	No
The Netherlands (*N* = 17.180000)	Standard communication system	GPS	1. Confirming the response 2. Location of victim, demographics (victim) and situational information	1. Get an AED from the vehicle; 2. Attach pads and follow the AED instructions; 3. Perform CPR; 4. Maintain a safe situation	1. AED; 2. Safety jacket; 3. Mobile phone/pager	Trained for CPR including use of an AED; Annually	None	No
Norway (*N* = 5.328212)	Standard communication system	Through communication with firefighter dispatch centre	1. Confirming the response 2. Location of victim; situational information	1. Get an AED from the vehicle; 2. Connect a breathing mask; 3. Attach pads and follow the AED instructions; 4. Perform CPR	1. AED; 2. Safety jacket; 3. Rescuer kit	Trained for CPR including use of an AED; Firefighters are motivated to train every year	None	No
Poland (*N* = 37.980000)	Standard communication system	Address	1. Confirming the response 2. Location of victim	1. Get an AED from the vehicle; 2. Connect a breathing mask; 3. Attach pads and follow the AED instructions; 4. Perform CPR	1. AED; 2. Mobile phone/pager; 3. Rescuer kit	Trained for CPR including use of an AED; *3 years*	NR	No
Portugal (*N* = 10.290000)	Standard communication system	Address	1. Using the radio 2. Location of victim; demographics; situational information	1. Get an AED from the vehicle; 2. Connect a breathing mask; 3. Attach pads and follow the AED instructions; 4. Perform CPR	1. AED; 2. Safety jacket; 3. Mobile phone/pager; 4. Rescuer kit	Training is not nationally organized, only in a few corporations firefighters are trained to use AED	NR	No
Romania (*N* = 19.530000)	Standard communication system; SMS; Pager	GPS; Address	1. Radio or phone report provide to the dispatch with physician on coordination 2. Location of victim; situational info	1. Get an AED from the vehicle; 2. Connect a breathing mask; 3. Attach pads and follow the AED instructions; 4. Perform CPR; 5. IV line; 6. Combi-tube	1. AED; 2. Safety jacket; 3. Mobile phone/pager; 4. Rescuer kit; 5. Valve mask; 6. IV; 7. Oxygen	Trained for CPR including use of an AED; *Every two years*	Firefighters receive a regular report of their activity/Designated EMS staff discuss calls attended with firefighters	Medical supplies, all materials for interventions; support for training and some equipment.
Slovenia (*N* = 2.084000)	Standard communication system	Location is not determined or tracked - they are activated if the ETA of the first responders is faster than the ETA of the EMS team - we expect them to be within the located district	1. Confirming the response 2. Location of victim	1. One person in the team has the AED/or AED at the station; 2. Connect a breathing mask; 3. Attach pads and follow the AED instructions; 4. Perform CPR	1. AED; 2. Mobile phone/pager; 3. Rescuer kit	Trained for CPR including use of an AED; *Annually*	Informally, e.g. reports on the results	Yes, not from the EMS but from the local municipality
Sweden, Norbotten County (*N* = 251,295)	Standard communication system; SMS	GPS	1. Confirming the response 2. Location of victim, demographics (victim)	1. Get an AED from the vehicle; 2. Attach pads and follow the AED instructions; 3. Perform CPR	1. AED; 2. Mobile phone/pager; 3. Rescuer kit	Trained for CPR including use of an AED; *Annually*	NR	No
Sweden, Region Western Sweden (*N* = 2.016000)	Standard communication system	GPS	1. Confirming the response 2. Location of victim, demographics (victim)	1. Get an AED from the vehicle; 2. Connect a breathing mask; 3. Attach pads and follow the AED instructions; 4. Perform CPR	1. AED; 2. Mobile phone/pager; 3. Rescuer kit	Trained for CPR including use of an AED; *Annually*	Meeting between EMS and fire fighters differs depending on municipality/sub region	No
Sweden, Stockholm (*N* = 965,232)	Standard communication system	GPS	1. The call is connected to the own dispatch centre whereby they confirm their response. 2. Location of victim & demographics (victim)	1. Get an AED from the vehicle; 2. Attach pads and follow the AED instructions; 3. Perform CPR	1. AED; 2. Mobile phone/pager; 3. Rescuer kit	Trained for CPR including use of an AED; *Annually*	Meeting a couple of times each year to discuss the collaboration	No
Switzerland, Ticino Canton) (*N* = 353,709)	SMS or APP	Not determined	1. Confirming the response 2. Location of victim, Location of AED, demographics (victim) & situational information	1. Get an AED from the vehicle; 2. Connect a breathing mask; 3. Attach pads and follow the AED instructions; 4. Perform CPR; 5. Assistance to bystanders; 6. Integration in advanced resuscitation (with ambulance team)	1. AED; 2. Safety jacket; 3. Mobile phone/pager; 4. Rescuer kit	Trained for CPR including use of an AED; *Bi-annually*	Designated EMS staff discuss calls attended with firefighters/Firefighters participate in a debrief with EMS staff immediately after an OHCA/Firefighters receive feedback based on AED data from the operational and clinical manager. They are invited to the annual ceremony, where survivors meet first responders	AED and rescue kit donated from Fondazione Ticino Cuore

If fire fighters are considered to be implemented in the total country, differences in density and characteristics, from city to city, may exist

*AED* Automatic External Defibrillator, *APP* (mobile) application, *BVM* Bag Valve Mask, *CPR* Cardiopulmonary resuscitation, *EMS* Emergency Medical Services, *ETA* Estimated Time of Arrival, *GPS* Global Positioning System, *OHCA* out of hospital cardiac arrest, *NR* Not reported; *SMS*, text message

**Table 2:** Characteristics of dispatched citizen-responders

Country, Region	Active system	Method to activate	Location determination	1. Response needed	Role onscene	Equipment	Training; Frequency	Registration	Feedback	Financial assistance	Emotional support	Other requirements to become a citizen-responder
				2. Information provided by dispatcher								
Austria (*N* = 8.220.000)	24 h/7	Standard communication system; SMS; Pager; APP	GPS	1. Confirming the response 2. Location of victim; location of AED	1. AED (private use); 2. Attach pads and follow AED instructions; 3. Perform CPR	1. AED; 2. Safety jacket; 3. Rescuer Kit	Yes trained for CPR including use of an AED; Annually	In an online system: Online App (for EMS, Paramedics, Medical Students, Physicians) or as designated First Responders (EMS/Paramedics) that are on call (during their free time)	Citizen-responders receive a regular report of their activity/Designated EMS staff discuss calls attended with citizen-responders	Yes: equipment and AED related disposables	Yes	A minimum of 18 years for the citizen-responders is required
Czech Republic Hradec Kralove Region (*N* = 550.804)	24 h/7	SMS; APP	GPS	1. Confirming the response 2. Location of victim; location of AED; situational information (Possibility to call dispatch centre for additional information)	1. Get a local AED; connect a breathing mask; 2. Attach pads and follow AED instructions; 3. Perform CPR Of note: Citizen-responders usually do not bring AEDs to the victim (density of local AEDs is low). They usually replace bystanders or relatives in doing high-quality CPR	1. Safety jacket; 2. Pocket mask, 3. Smartphone	Yes trained for CPR including use of an AED; Any valid ERC course certificate is needed	In an online system: SW KISS SHARP (new name: O2 SOS), registration and information at fr.zzskhk.cz	Letter of thanks	Yes: safety vest, pocket mask, until 2017 also smartphone	Yes, critical incident stress management available on request	A minimum of 18 years for the citizen-responders is required and they must declare to have no criminal history. This must be proven by an official document issued by the state-wide registry.
Denmark, Capital region, Central Region (*N* = 3.137.000)	24 h/7	APP	GPS	1. Confirming the response 2. Location of victim; location of nearest accessible AED	1. Get a local AED; 2. Attach pads and follow AED instructions; 3. Perform CPR	1. AED	Not required but strongly recommended	In an online system: In the app server	Via mail and social media when asked for	No	Yes, critical incident stress management available on request	A minimum of 18 years for the citizen-responders is required
Denmark, Region Zealand, Region South (*N* = 2.056.025)	24 h/7	APP	GPS	1. Confirming the response 2. Location of victim; location of AED	1. Get a local AED; 2. Attach pads and follow AED instructions; 3. Perform CPR; 4. If the citizen responder wants: guide the ambulance to the right address and assist the ambulance personnel.	1. AED	Yes trained for CPR including use of an AED; at least a 4 h CPR training course	In an online system: In the app server	Via mail and social media when asked for	No	Yes, critical incident stress management available on request	A minimum of 18 years for the citizen-responders is required and absence of a criminal history regarding child abuse
England^a^ (*N* = 55.980000)	24 h/7	APP	GPS and Address	1. Confirming the response 2. Location of victim; demographics (victim) and situational information	1. Get an AED out of the vehicle; 2. Connect a breathing mask; 3. Attach pads and follow AED instructions; 4. Perform CPR; 5. They are trained in airway management	1. AED; 2. Rescuer Kit	Yes trained for CPR including use of an AED; Annually	In an online system: In house database	Citizen-response activity is described in the annual report/Citizen-responders receive a regular report of their activity/Citizen-responders receive written feedback on individual calls from the EMS	No	Yes	Each Trust sets its own parameters. Most require a minimum of 16 or 18 years for the citizen-responders. Also, the criminal record is checked.
Germany^b^, Marburg-Biedenkopf, Gütersloh, Freiburg, Neckar-Odenwald-Kreis, Greifswald, Schleswig-Holstein (*N* = 3.661.774)	24/7	SMS; APP	GPS	1. Confirming the response 2. Location of victim; location of AED	1. Get a local AED; 2. Connect a breathing mask; 3. Attach pads and follow AED instructions; 4. Perform CPR	1. AED; 2. Safety jacket; 3. Rescuer Kit	Yes trained for CPR including use of an AED; Annually	In an online system: In the app server/German Red Cross	No	No	Yes, they can choose to contact a special team for debriefing	A minimum age of 18 years for the responders is required
Ireland (*N* = 4.830.000)	24 h/7	SMS	Not determined	1. Confirming the response 2. Location of victim; location of AED; demographics (victim) and situational information	1. Get a local AED or get an AED out of the vehicle; 2. Connect a breathing mask; 3. Attach pads and follow AED instructions; 4. Perform CPR	1. AED; 2. Safety jacket; 3. Resuscitation kit bag	Yes trained for CPR including use of an AED; Monthly	Authorisation granted by Ambulance Officer	Citizen-response activity is described in the annual report/Citizen-responders receive a regular report of their activity/Designated EMS staff discuss calls attended with citizen-responders	Replacement of consumable equipment	Yes	A minimum age of 18 years for the citizen-responders is required and they must get Garda Clearance, i.e., confirmation must be received from the Irish police that the person does not have a criminal conviction of significance
Italy Emilia Romagna (*N* = 4.453.000)	24 h/7	APP	GPS	1. Confirming the response 2. Location of victim; location of AED	1. Get a local AED; connect a breathing mask; 2. Attach pads and follow AED instructions; 3. Perform CPR	None	Yes trained for CPR including use of an AED; Bi-annually	In an online system: https://www.118er.it/dae/	The responder manager sends an email to collect feedback by mail (but is not a routine)	No	No	No requirements
Hungary (*N* = 9.800.000)	24 h/7	Standard communication system; APP	GPS	1. One person from the dispatch centre calls the citizen to check whether she/he is able to go to the victim 2. Location of victim; location of AED	1. Get a local AED; 2. Attach pads and follow AED instructions; 3. Perform CPR	1. AED	Not required; There is not any training for them, it depends on the citizen.	In an online system: Szív city (szivcity.hu)	None	No	No	No requirements
The Netherlands Except Amsterdam (*N* = 15.835350)	24 h/7	SMS; APP	GPS; Address	1. No response is required 2. Location of victim; location of AED	1. Get a local AED or get an AED out of the vehicle; 2. Attach pads and follow AED instructions; 3. Perform CPR	1. AED	Yes trained for CPR including use of an AED; Annually	In an online system: Hartslag.nu	None	No	Hartslag.nu asks for an evaluation after the event	A minimum age of 18 years is required and no dispatch to OHCAs caused by a trauma or in children < 8 years
Romania, Bucharest (*N* = 1.828.000)	NR	SMS	GPS	1. No response is required 2. Location of victim; location of AED	1. Get a local AED; 2. Attach pads and follow AED instructions; 3. Perform CPR	1. AED	Yes trained for CPR including use of an AED; Only trained once	NR	Activity is described in the annual report	No	Informal emotional support from Ambulance Service Staff	A minimum age of 16 years for the citizen-responders is required
Sweden, Norrbotten County (*N* = 251.295)	24 h/7	SMS	Triangulation by telephone towers	1. No response is required 2. Location of victim	1. Perform CPR	None	Yes trained for CPR including use of an AED; Responders should be educated but no specific follow up is done	In an online system: https://www.mobilraddare.se/	NR	Yes, please describe the type of assistance given: not specified	Yes, if requested they can contact the project via email/telephone	A minimum age of 18 years is required and no dispatch to children < 8 years
Sweden, Region western Sweden (*N* = 2.016.000)	During daytime: 07:00–22:59	SMS	GPS	1. Confirming the response 2. Location of victim; location of AED	1. Get a local AED or get an AED out of the vehicle; 2. Attach pads and follow AED instructions; 3. Perform CPR	None	Yes trained for CPR including use of an AED; All should be trained once. The repetitions courses differs between individuals	Directly in the APP	None	No	Yes, crisis consultation if requested.	A minimum age of 18 years for the citizen-responders is required and no dispatch to children < 8 years
Sweden, Stockholm (*N* = 965.232)	During daytime: 07:00–22:59	SMS; APP	GPS	1. Confirming the response 2. Location of victim; location of AED and situational information	1. Get a local AED; 2. Attach pads and follow AED instructions; 3. Perform CPR	None	Yes trained for CPR including use of an AED; Only trained once	In an online system: Register directly via application	Via mail and social media when asked for	No	Yes, debriefing if needed (if requested crisis counselling is given up to 10 visits)	A minimum age of 18 years is required and no dispatch to children < 8 years
Scotland (*N* = 543.800)	Book on with the control centre when available. Therefore, their coverage varies across the country at any given time	Standard communication system	GPS	1. Confirming the response 2. Location of victim; situational information	1. Get an AED out of the vehicle; 2. Connect a breathing mask; 3. Attach pads and follow AED instructions; 4. Perform CPR; 5. Insert airway if required	1. AED; 2. Safety jacket; 3. Rescuer Kit	Yes trained for CPR including use of an AED; Community First Responders go through an initial 4 day training course and then are expected to attend monthly training sessions within their groups, which follow a structured programme	In an online system: After training and suitability checks have been completed they are put on a database and classed as an active responder) and then when available they book on and ACC colleagues can deploy them	Citizen-response activity is described in the annual report; Designated EMS staff discuss calls attended with citizen-responders/Citizen-responders receive written feedback on individual calls from the EMS/We have a network of Community Resilience Facilitators who meet with Community First Responder groups periodically and are available to support them	Yes: the can claim mileage costs if they have used their own vehicle. Scottish Ambulance Service also replaces any consumable items used during the course of the first responders attendance	Yes	A minimum age of 18 years for the citizen-responders is required and dispatch to adults only; all citizen-responders undergo a protection of vulnerable groups (PVG) check and, depending on feedback, a judgement is made on the suitability to be a citizen-responder.
Switzerland, Ticino Canton (*N* = 353.709)	24 h/7	APP	Not determined	1. Confirming the response 2. Location of victim; location of AED; demographic and situational information	1. Get a local AED; 2. Connect a breathing mask	1. Safety jacket	Yes trained for CPR including use of an AED; Bi-annually	In an online system: smartphone Application (MOMENTUM) and registration program created by us (fr.ticinocuore.ch)	Receive written feedback on individual calls from the EMS/ Participate in a debrief with EMS staff immediately after an OHCA/ Receive feedback based on AED data from the operational and clinical manager/Are invited to the annual ceremony, where survivors meet first responders	No	Yes, 24 h/7 days team of psychological counsellors	A minimum age of 18 years is required (of note: citizens are allowed to use an AED from age 14 years). Citizen-responders must adhere to the general conditions. They need to self-declare absence of a criminal record, but declarations are not checked.
Switzerland, Region of Fribourg (*N* = 225.500)	24 h/7	APP	GPS	1. Confirming the response 2. Location of victim; location of AED; demographics (victim) and situational information	1. Get a local AED	1. Safety jacket	Yes trained for CPR including use of an AED; Every two years	In an online system: Citizen responders must create an account in the app	Citizen-responder must complete a feedback sheet. Dependent of the answers, the foundation will contact the responder by phone. Sometimes citizen-responders can have immediately feedback from the EMS staff.	Yes, the foundation pay the replacement of AED consumable after intervention	Yes, citizen-responders have the possibility to call a psychological service	A minimum age of 18 years is required (of note: citizens are allowed to use an AED from age 14 years). Citizen-responders must adhere to the general conditions. They need to self-declare absence of a criminal record, but declarations are not checked.

If citizen-responders are considered to be implemented in the total country, differences in density and characteristics, from city to city, may exist

Abbreviations: as in Table 1

^a^The respondent specifically indicated that APP usage is not universal and (major) differences across the country exist

^b^There are four different APP-systems in Germany. Characteristics apply to region Marburg-Biedenkopf but may differ across the other regions

**Table 3:** Characteristics of dispatched police officers

Country, Region	Activation by	Location determination	1. Response needed	Role on scene (steps)	Equipment	Training, Frequency	Feedback	Financial assistance (NOT including: salary)	Other
			2. Information provided by dispatcher						
Austria (*N* = 8.220000)	Standard communication system; SMS; Pager; App	GPS	1. Confirming the response 2. ocation of victim; location of AED (if applicable)	1. Get a local AED or from the vehicle; 2. Attach pads and follow the AED instructions; 3. Perform CPR	1. AED; 2. Safety jacket; 3. Mobile phone/Pager; 4. Rescuer kit	Yes trained for CPR including use of an AED; Annually	Police officers receive a regular report of their activity	AED equipment and related disposables	
Belgium, Brussels (*N* = 1.208500)	Standard communication system	GPS	1. Confirming the response 2. Location of victim	1. Get an AED from the vehicle; 2. Attach pads and follow the AED instructions; 3. Perform CPR	1. AED	Yes trained for CPR including use of an AED; Variable frequency (depends on police district)	There is no feedback to the officer’s superiors, after the intervention they are debriefed as part of the EMS team, at their demand.	NR	
Czech Republic, (*N* = 10.521646)	Standard communication system	GPS and Address	1. Subsequent call from dispatcher to police officer/ backwards 2. Location of victim; demographics (victim) and situational information	1. Get an AED from the vehicle; 2. Connect a breathing mask; 3. Attach pads and follow the AED instructions; 4. Perform CPR	1. AED; 2. Safety jacket; 3. Mobile phone/Pager; 4. BVM without oxygen	Yes trained for CPR including use of an AED; Annually	Designated EMS staff discuss calls attended with firefighters/ Police officers participate in a debrief with EMS staff immediately after an OHCA/ Regular meetings with police representatives/ EMS quality managers can take part if urgent problems need to be solved	AEDs located in some police cars are purchased by EMS	Police stations are usually located at places where no ambulance stations are
England (*N* = 55.980000)	APP	Address	1. Confirming the response 2. Location of victim; demographics (victim) and situational information	1. Get an AED from the vehicle; 2. Connect a breathing mask; 3. Attach pads and follow the AED instructions; 4. Perform CPR; 5. Trained in airway management	1.AED; 2. Mobile phone/Pager; 3. Rescuer kit	Yes trained for CPR including use of an AED; Annually	Police officer activity is described in the annual report/ Police officers receive a regular report of their activity; Police officers receive written feedback on individual calls from the EMS/ Police officers participate in a debrief with EMS staff immediately after an OHCA	No	
Ireland (*N* = 4.830000)	SMS	Not determined	1. Confirming the response 2. Location of victim; location of AED; demographics (victim)	1. Get a local AED or from the vehicle or PAD; 2. Connect a breathing mask; 3. Attach pads and follow the AED instructions; 4. Perform CPR	1. AED; 2. Safety jacket; 3. Mobile phone/Pager	Yes trained for CPR including use of an AED; Annually	Police officer activity is described in the annual report/ Police officers receive a regular report of their activity/ Police officers participate in a debrief with EMS staff immediately after an OHCA	Consumable equipment replacement	
Italy, Emilia Romagna (*N* = 445,300)	App	GPS	1. Confirming the response 2. Location of victim; Location of AED (if applicable)	1. Get a local AED or from the vehicle; 2. Connect a breathing mask; 3. Attach pads and follow the AED instructions; 4. Perform CPR	1. AED; 2. Safety jacket; 3. Mobile phone/Pager; 4. Rescuer kit	Yes trained for CPR including use of an AED; Bi-annually	The responder manager sends an email to collect feedback by mail (but is not a routine)	No	
The Netherlands (*N* = 17.180000)	Standard communication system	GPS	1. Confirming the response 2. Location of victim; demographics (victim)	1. Get an AED from the vehicle; 2. Attach pads and follow the AED instructions; 3. Perform CPR; 4. Do safety measures and provide a safe workspace for people involved	1. AED; 2. Safety jacket;	Yes trained for CPR including use of an AED; Annually	None	No	
Sweden, Stockholm (*N* = 965,232)	Standard communication system	GPS	1. Police is dispatched by their own dispatch centre. If available they answer by oral communication 2. Location of victim; demographics (victim)	NR	1.AED; 2. Mobile phone/Pager	Yes trained for CPR including use of an AED	Meetings a couple of times each year where they discuss the collaboration	No	
Switzerland, region of Fribourg (*N* = 225,500)	App; In case of OCHA, EMS dispatch centre calls the police dispatch centre to give the alarm. Police men can receive alarms from the police dispatch or from the app (like other first responders)	GPS	1. Confirming the response 2. Location of victim	1. Get an AED from the vehicle; 2. Attach pads and follow the AED instructions; 3. Perform CPR	1. AED; 2. Safety jacket; 3. Mobile phone/Pager	Yes trained for CPR including use of an AED; Every 3 years	Police officers receive sometimes feedback but it is not systematic	Yes: AED Electrodes are paid by the first responders foundation	
Switzerland, Ticino Canton (*N* = 353,709)	APP	Not determined	1. Confirming the response 2. Location of victim; location of AED; demographics (victim) and situational information	1. Get an AED from the vehicle; 2. Connect a breathing mask; 3. Attach pads and follow the AED instructions; 4. Perform CPR; assistance to bystanders; 5. Integration in advanced resuscitation (with ambulance team)	1.AED; 2. Safety jacket; 3. Mobile phone/Pager; 4. Rescuer kit	Yes trained for CPR including use of an AED; Bi-annually	Designated EMS staff discuss calls attended with police officer/ Police officers receive written feedback on individual calls from the EMS/ Police officers participate in a debrief with EMS staff immediately after an OHCA/ Police officers receive feedback based on AED data from me. they are invited to the annual ceremony, where survivors meet first responders	Yes: AED and rescue kit donated free from Ticino Cure Foundation	

If police officers are considered to be implemented in the total country, differences in density and characteristics, from city to city, may exist

Abbreviations: as in Table 1

#### Response characteristics: availability and alerts

Different methods were used to alert FRs. For firefighters and police officers, a standard communication system is often used. A smaller proportion of regions used a dedicated mobile phone alert (Tables 1 and 3).

Citizen-responders are dispatched using a dedicated mobile phone alert in all but one region (in which only the standard communication system is used). While firefighters and police officers tend to be available on a 24/7 basis, this is not the case for all FR types (Table 2).

In several countries, there is an age threshold to be dispatched as a citizen-responder (e.g., ≥16 or ≥ 18 years). Also, in a few countries there is no dispatch of citizen-responders to children (e.g., < 8 years).

#### Equipment

In every country and region, FRs either carry an AED, or are directed by the dispatch centre to the nearest publicly accessible AED. Safety jackets, pocket masks, mobile phones, and rescuer kits are generally part of the equipment.

#### Training and registration

In most European regions, CPR training is required and checked before FRs can be dispatched, except for citizen-responders in Denmark (in two regions: Capital region and Central region) and Hungary. The frequency of mandatory CPR training differed between countries, particularly for citizen-responders (varying from monthly training to none). In Italy, untrained citizens are by law not allowed to use an AED, but can perform CPR.

Citizen-responders are most commonly registered in online databases such as HartslagNu (the Netherlands), MOMENTUM (Switzerland), O2 SOS (Czech Republic), DAE respondER (Italy). In some countries, including Ireland and Scotland, registration is managed by the ambulance service on an EMS-owned database. Several countries (Denmark: region Zealand and Southern Denmark, Czech Republic, Ireland and Switzerland) require absence of a criminal record (or of a criminal conviction of significance) in order to be able to register as a citizen-responder.

### National and regional policies

Policies relating to the implementation of FR-systems are described on national or regional level, or both (Table 4). National policies may apply to the total FR-system or may be limited to one FR type only (Additional file 1: Table S2).

**Table 4:** Analysis of national policies relating to First Responders, per country

Country	Implementation: National or Regional	Short description of policy
Austria	Regional	Not reported
Czech Republic	National and regional	There is a national policy related to professional FRs (firefighters/police officers). This policy is very general (e.g. CPR training requirement). All 14 regional EMS organizations in the country have been using professional FRs to some extent. They differ a little across the areas to optimise the best strategy per region. Only 1 EMS has also introduced a mobile app for alerting FRs, incl. Both off-duty EMS personnel and citizen FRs. Citizen FRs need to have a valid BLS/AED course certificate.
Denmark	National and regional	Denmark has a strategy of engaging the community in saving lives after OHCA and succeeded in tripling the bystander rate and the survival after OHCA within the last 15 years. New programs dispatching citizen-responders through a smart phone application has been implemented within the last few years. The government of Denmark supports the 10 steps of increasing survival after OHCA defined by the Global Resuscitation Alliance, GRA: https://www.globalresuscitationalliance.org/wp-content/pdf/acting_on_the_call.pdf and has a national “Resuscitation Academy” program working to implement the 10 steps in the five Danish regional EMS organizations. Denmark also has some experience including professional fire fighters and police at OHCA already and expects to strengthen this collaboration within the next years.
Ireland	National and regional	The National Ambulance Service has a history of FR involvement which predates the 2000s. General practitioners have been acting as FRs in selected parts of the country since the early 1990s. The first formal recommendation to support the development of first responders appears in the following national document which was produced by our Department of Health: “Reducing the Risk: A Strategic Approach. Report of the Task Force on Sudden Cardiac Death (2006)”. The National Ambulance Service has policy and procedure documents to support first responder involvement in cardiac arrest response. Ireland is in the process of developing a national OHCA strategy, which will include further specific recommendations to further the development of first response in Ireland.
Italy	Regional	Law in Italy: Citizen trained in BLS are allowed to start CPR and to use an AED; citizens not trained can start CPR and use an AED accordingly to the law that regulates the “state of necessity” in emergency situations. The law in Italy for FRs and untrained lay people is a barrier to diffusion of bystander intervention. Some regions in Italy (Region of Emilia Romagna) implemented FRs. FRs will become more common in Italy in other regions (e.g., Province of Pavia).
Luxembourg	Regional	Since July 1, 2018 Luxembourg is completely reorganised in only one EMS organisation for the country. Luxembourg has a FR system working in 56 out of 102 municipalities. This service is provided exclusively by volunteer firefighters because they are “in the system” and thus quite easily reachable. There are different modalities according to local circumstances. Either the FR get to the scene by their own means (when they have the equipment in their car) or they meet at the fire station to get their equipment before going to the scene. In Luxembourg, for the moment, there are only firefighters acting as “organized” FRs. Police is more reluctant to participate and there is no citizen-responder system. There is a mandatory training in CPR for school children since 2017 and the Luxembourg Resuscitation Council strives to train as many as possible persons in elementary CPR (hands-only) on a voluntary basis.
Netherlands	National and regional	In the Netherlands, a national policy regarding citizen-responders was published, whereas the requirements for firefighters and police officers are described on regional level only. Regarding the implementation of AEDs and citizen-responders, the first policy was written in 2002. The reason to involve no citizen-responders in the capital Amsterdam is the proximity of police officers and firefighters (always shorter than 6 min to OHCA victim).
Norway	National and regional	The Directory of health is working on policies and guidelines for FRs, including what a FR is, who can be called a FR and legal implications and concerns. There are 16 dispatch centres in Norway and they are all relating to firefighters as FRs in different ways
Portugal	Regional	At the present moment, private investors are spreading AED programs with the main purpose to have a system with FRs in the event of an OHCA. In one region (21.000 people of 506.000) of Lisbon, police officers have been trained in BLS and AED. Portugal is in a preliminary process regarding the start of FRs.
Slovenia	National and regional	In Slovenia, only voluntary fire fighters are implemented as FRs. This is due to a very extended network of firefighters brigades in Slovenia (literally, every village in Slovenia has a voluntary firefighters brigade). At the present moment, 35.000 volunteer fire fighters are organized in approximately 1500 fire fighters brigades. This number exceeds the number of policemen by six-times (approximately 5500 policemen). Voluntary firefighters in Slovenia are well organized and equipped (vehicles, communication, rescue equipment, etc.). On the other hand there is no nationwide strategy “how to organize FRs”. The Slovenian government released the document/regulation which basic conditions must be fulfilled to become a FR (skills, equipment, etc.). But the organization of FR is left to the local EMS. The local EMS firstly considers the need of first responders (especially in remote areas). Afterwards the local fire fighters brigade will be contacted to participate in a FR system (there is no obligation). Therefore, there are areas in Slovenia without FRs. Policemen patrol network is scarce compared to the fire fighters (there is interest by policemen to be also a part of a FR system).
England	National and regional	In England, ambulance trusts are responsible for local implementation, but there is an overarching Governance Framework.
Sweden	Regional based	Strategies or policies recommending establishment or the development of FRs in Sweden is lacking. Policies are regional.
Switzerland	Regional based	In Switzerland all health issues (except the management of epidemics and disasters) are left to the cantons. It follows that with 26 cantons, there are 26 different health laws. Since the FR network is still considered ancillary to the EMS, there is no health law that takes this into account. This is the reason why there are so many different approaches (even inside the cantons). Only a few cantons have created a structure for the purpose of uniformly managing the FRs. Leading position and pioneer is Canton Ticino (https://www.ticinocuore.ch/en). The Swiss Resuscitation Council is working on a national strategy against OHCA.

*AED* Automatic External Defibrillator*, CPR* Cardiopulmonary Resuscitation*, EMS* Emergency Medical Services*, ERC* European Resuscitation Council*, FR* First-responder*, OHCA* Out Of Hospital Cardiac Arrest

### Future implementations of FR-systems in Europe

As shown in Fig. 2b, some countries did not dispatch FRs to attend an OHCA at the time of the survey. Respondents from Bosnia-Herzegovina, Croatia, Cyprus, Estonia, Greece, France, Iceland, and Serbia were not aware of plans to introduce FR-systems to their countries in the short term. Specific reasons for this are described in Additional file 1: Table S2, and include: (1) implementation of FRs is not a subject of interest or not considered as a priority; (2) there is a lack of a legal background definition for FRs, and (3) there are some local unmapped AEDs, but the location of these AEDs is not available to the dispatch centre.

At the time of our survey, in Malta, government and non-governmental organisations were negotiating to implement a FR-system. In Spain, at least two regional EMS-systems were recruiting citizens to respond to OHCA. In Italy, there were indications that the Province of Pavia would begin implementing FR-systems within a few months (Additional file 1: Table S2).

## Discussion

### Key findings

Our study shows the variety of FR-systems that have developed in Europe to expedite provision of good quality CPR and defibrillation in case of an OHCA. These FR-systems have either been implemented nationwide or regionally, and development is primarily influenced by local initiatives, circumstances and opportunities. Policies regarding FRs are commonly implemented on a regional level, even if a national policy exists. This has resulted in a wide variety of FR-systems both *between* and *within* countries. Even in countries that do not have FR-systems in place, local and national initiatives to implement FR-systems are being developed.

### The need for FR-systems

The evidence for the benefits of early defibrillation are clear, therefore it may be suggested that increasing the number of AEDs available should be sufficient to improve OHCA survival. However, an increased number of AEDs alone is unlikely to improve survival in a cost-effective manner, as demonstrated by an Irish Health Technology Assessment which calculated that an investment of €105 million in AED purchase would yield – at best – an additional 10 lives saved per year [23]. Rather, the strategic deployment of AEDs by CPR-competent FRs may be an important link in the Chain of Survival [12, 16, 17], as acknowledged in the most recent European Resuscitation Guidelines [18].

We observed that, even in countries with a national FR-policy, the organisation of FR-systems is often managed by regional EMS. The design of FR-systems is thus commonly influenced by local circumstances and by what is available. For instance, in Slovenia, a FR-system with voluntary firefighters was chosen because of the extensive network of volunteer fire brigades across all villages. Although there is interest from police officers to be part of this FR-system, the network of police patrols in Slovenia is less dense than that of fire fighters and, therefore, police are not included in the FR-system.

### Important aspects of FR-systems

It is unlikely that a “one size fits all” FR-system in Europe can be implemented. However, by combining results from this study with previous studies, some important aspects of FR-systems have been identified.

First, FR type and number of dispatched FR types within one FR-system may be important. In our study, firefighters featured highly as FR types and previous research has demonstrated their role in OHCA-survival [11, 12, 24, 25]. FR-systems involving police officers and/or dispatched citizen-responders are very promising, but more research is needed [14, 26, 27]. In certain regions, multiple FR types in one FR-system exist. So far, only limited evidence towards the effectiveness of having multiple FR-types is available. A study performed in the Netherlands by Zijlstra and colleagues showed that, while the contribution of citizen-responders was limited by the strong involvement of other FRs and their competing contribution to OHCA care, it was estimated that, without the citizen-responders, 7.3% of patients would not have received a first shock within 6 min [16]. Also, in Sweden, a study comparing additional dispatch of CPR trained firefighters and police officers equipped with AEDs to a control group where only EMS was dispatched showed that dispatching these two FRs was associated with a significant increase in 30-day survival [27]. However, more research is needed.

Second, the method of alerting FRs matters. Our results showed that firefighter and police FRs are commonly alerted by their own standard dispatch system, and previous evidence highlighted the benefit of direct communication between the EMS and firefighter and police FRs [28, 29]. A mobile phone alert is often used to alert citizen-responders, but only a few regions use a mobile phone alert to alert other FR types (i.e., firefighters and police officers). In a study carried out in Switzerland, all FR types (firefighters, police, citizen-responders) were alerted by either an app or text-message system (both considered as a mobile phone alert) [15]. The app-system, when compared to a text-message system, was found to be highly efficient in the deployment of FRs, significantly reducing the time to initiation of CPR and increasing survival rates [15].

Third, our study showed that the response capabilities of FRs should be considered. In the Czech Republic, firefighters were dispatched only in rare cases because they share locations with the EMS. Another example regarding response capabilities includes: in several countries there is no dispatch to children < 8 years by citizen-responders. Although this applies to a minority of the OHCA population, it should be taken into account. Also, the distinction between volunteer and professional FRs may have an impact on FR engagement and response. Another example includes Slovenia where the local EMS determines the need for FRs, and requests local volunteer firefighters to become FRs. As this strategy depends on local firefighter interest, there are still areas in Slovenia without FRs.

Fourth, our study showed that frequent CPR training is a feature of most FR-systems, as would be expected. Previous research has highlighted the superiority of off-duty medical professionals over laypersons [30] and more recent studies have shown the positive impact of trained citizen-responders on neurological outcomes [31]. Some regions allow citizen-responders to register as FRs without validating CPR-training (e.g., Denmark: Capital region and Central region), whereas CPR training is mandatory in two other regions in Denmark (Region Zealand and Region Southern Denmark). However, in the two regions where CPR training is not mandatory, CPR training is strongly recommended. Also, in Denmark, large-scale population-based CPR training is common, and the positive impact of population-based CPR training has most recently been demonstrated by Kobayashi and colleagues in Japan [32]. Finally, improved survival after implementation of FR-systems is unlikely to occur unless all links in the Chain of Survival are working. Hence, improvements in bystander-CPR should receive high priority.

### Future implementation of FR-systems

We hypothesise that the general tendency in Europe towards more widespread implementation of FR-systems will increase OHCA survival rates. At present, FR-implementation may not be a priority for every country and difficulties in the legal definition of FRs may contribute to this. However, lack of national policies may not be an impediment to local development. For instance, in Greece, small local initiatives already exist in the absence of a national policy. In Croatia, local initiatives are developed, including nurses on motorbikes and CPR-trained firefighters and police officers equipped with AEDs. However, these initiatives are not currently connected to the EMS to be dispatched and this may limit their rapid response. In other countries and regions, while there is interest, local AEDs are not registered; this may also delay implementation of an effective FR-system. These developments highlight that FR-systems are strongly driven by local initiative and local capabilities. The need to allow flexibility in how FR-systems are implemented locally is likely to be an important consideration in ensuring the sustainability of FR-systems into the future.

### Strengths and limitations

To the best of our knowledge, this study provides the most comprehensive overview of first response in Europe to date. While heterogeneity in FR-systems is a key finding, common themes have been identified that provide a basis for understanding the development of FR-systems at a European level. It is acknowledged that a convenience sampling method was used to recruit respondents, but the sample was drawn from participants in well-established European networks that have an active interest in OHCA.

A limitation of this study is that, in countries/regions considered as being covered by a specific FR-system, it was not feasible to estimate the density of FRs (and differences, from city to city, may exist). Also, it was not possible to relate different FR-systems to differences in survival rates. However, this study has highlighted a unique element of the Chain of Survival which should be considered in any further studies of OHCA epidemiology.

When a FR-system is being developed, it is important to analyse response times in order to confirm that the system actually contributes to early CPR and early defibrillation, using measured effects. Only then in the long run, the cost and effort of maintaining such a FR-system will remain accepted in the community.

## Conclusions

At present, more than half of European countries dispatch FRs after a suspected OHCA. Policies relating to FRs are mostly managed by local EMS, leading to a wide variation *between* and *within* countries. Even in countries that do not have existing FR-systems, many have local initiatives and future plans for FR-system implementation. The willingness of people to volunteer their time and skills to provide a first response to OHCA has led to the development of a variety of national and local solutions, and has created a new paradigm within the Chain of Survival that needs to be researched and evaluated more extensively. Areas for future research include: identifying the most effective methods of FR dispatch; identifying FR-systems that most strongly influence survival; assessing the effect of adding a FR type in an existing FR-system; and understanding what motivates a volunteer to become a FR and what sustains that motivation. The diverse findings of our study reflect the diversity in circumstances across various European regions and suggest that it is unlikely that there will be a ‘one size fits all’ FR-system across Europe. Rather, an overall European policy that advises on the critical requirements for effective FR may be of benefit.

## Supplementary information

**Additional file 1.** Respondents and survey definitions.

## Data Availability

The datasets used and/or analysed during the current study are available from the corresponding author on reasonable request.

## Abbreviations

AED: automated external defibrillator
ARREST: Amsterdam Resuscitation Studies
CPR: Cardiopulmonary resuscitation
EMS: emergency medical service
ERC: European Resuscitation Council
FR: first responder
OHCA: out-of-hospital cardiac arrest
