# Supplementary material for: First-response treatment after out-of-hospital cardiac arrest: a survey of current practices across 29 countries in Europe
Source: Scand J Trauma Resusc Emerg Med. 2019 Dec 16;27:112. doi: 10.1186/s13049-019-0689-0 (PMC6916130; doi:10.1186/s13049-019-0689-0)
Supplement: Supplementary file 1 — Additional file 1. Respondents and survey definitions. [file 13049_2019_689_MOESM1_ESM.docx]

**Supplementary 1a, Additional information (survey) respondents**

*General information of respondents*

Among the survey respondents, 20 EuReCa^2^ National Coordinators participated. Additionally, 15 ESCAPE-NET^19^ members and 16 other European Experts in the field of OHCA resuscitation completed the survey.

|  | Number of respondents to survey | Additional information from resuscitation council or from other expert in the field* |
| --- | --- | --- |
| Austria | 1 | Yes |
| Belgium | 3 | Yes |
| Bosnia-Herzegovina | 1 | Yes |
| Croatia | 1 | Yes |
| Cyprus | 1 | Yes |
| Czech Republic | 1 | Yes |
| Denmark | 2 | Yes |
| Estonia | 1 | Yes |
| Finland | 1 | No |
| France | 2 | Yes |
| Germany | 3 | Yes |
| Greece | 1 | Yes |
| Hungary | 2 | Yes |
| Iceland | 3 | No |
| Italy | 2 | Yes |
| Ireland | 1 | Yes |
| Luxembourg | 2 | Yes |
| Malta | 2 | Yes |
| The Netherlands | 3 | Yes |
| Norway | 1 | Yes |
| Poland | 1 | No |
| Portugal | 4 | Yes |
| Romania | 1 | Yes |
| Serbia | 1 | Yes |
| Slovenia | 1 | Yes |
| Spain | 3 | Yes |
| Sweden | 1 (completed 5 surveys for different regions) | Yes |
| Switzerland | 4 | Yes |
| United Kingdom | 2 | Yes |

**Additional information: policy information*

**Supplementary 1b, Definitions of Emergency Medical Services and First-Responders**

**Emergency Medical Service (EMS)**

An EMS is defined as a service dedicated to providing out-of-hospital acute medical care. For this survey, EMS includes ambulance or responding vehicle, and/or helicopter services dispatched by a dispatch centre to provide acute medical care and to transport the patient to a hospital equipped to provide acute care.

**First-Responder (FR)**

In 2000, the American Heart Association and the International Liaison Committee on Resuscitation defined three levels of responders^22^. Level one is ‘Traditional Responders’ and includes those whose role it is to respond in an emergency, e.g., police officers and fire fighters. Level two are ‘Citizen-responders’, and level three are responders to those at high risk (e.g., family members of high-risk patients). For this study, the definition of FRs includes level one and two responders, but is limited to individuals who are dispatched to the event. In summary, for the purposes of this study, FRs are defined as all individuals who are *not* on-duty members of the EMS and are *dispatched* by a dispatch centre to attend OHCA events.

Dispatched FR treatment potentially included:

- Members of the general population who respond independently or as part of a community-based citizen-responder group which is linked to the ambulance service
- Firefighters or police officers;
- Off-duty EMS staff;
- Voluntary and auxiliary services that are not paid to be on-duty with the EMS at the time of the OHCA event.

Dispatched FR treatment does not include:

- On-duty ambulance staff, regardless of whether they were alerted to the scene or arrived on scene by chance (e.g., when they came across an OHCA event *en route* to another call);
- Any individual who arrived on scene by chance, regardless of his/her level of expertise.

**Supplementary 1c, Characteristics of the First-Responder systems, as used in the survey**

**Response characteristics**

Response characteristics were defined as follows:

1. Method to activate the First-Responder (FR):
2. By a dedicated communication system (Yes/No);
3. By text-message (SMS) (Yes/No);
4. By pager (Yes/No);
5. By app (Yes/No);
6. Other, specified (Yes/No).
7. Location determination:
8. By GPS (Yes/No);
9. By address (Yes/No);
10. Not determined (Yes/No).
11. Time threshold in minutes and distance in km to victim;
12. Whether a response of the FR to the dispatcher is required:
13. Yes, by pressing the button;
14. No response is required;
15. Other.
16. Information communication between dispatcher and FR:
    - 1. Location of the victim (Yes/No);
      2. Location of an AED (Yes/No);
      3. Demographic information victim (Yes/No);
      4. Situational information (Yes/No);
      5. Other (Yes/No).
17. For the citizen-responders, information on whether they were dispatched manually or automatically was retrieved. An automatic dispatch of the citizen-responder includes no involvement of the dispatcher after a notification of a suspected cardiac arrest. A manual dispatch includes involvement of the dispatcher.

**Role on scene, equipment, training and registration**

1. Regarding the role on scene, information was retrieved on what would be applicable for the FR when arriving at the victim :
2. Get a local AED (Yes/No);
3. Get an AED out of the vehicle (Yes/No);
4. Connect a breathing mask (Yes/No);
5. Attach pads and follow AED instructions (Yes/No);
6. Perform CPR (Yes/No);
7. The FR is not allowed to start CPR and/or use an AED (Yes/No);
8. Other (Yes/No).
9. Equipment that is available to the FR:
10. AED (Yes/No);
11. Safety jacket (Yes/No);
12. Rescue kit (Yes/No);
13. Other (Yes/No).
14. Training:
15. CPR trained including use of an AED and frequency of the training
16. Not CPR trained including use of an AED
17. For the citizen-responders, the place of registration is retrieved
18. In an online system (specified)
19. Not registered
20. Other.

**Feedback, financial and emotional support**

1. Style of feedback provided to the FR:
2. FR is described in the annual report (Yes/No);
3. FR receive a regular report of their activity (Yes/No);
4. Designated EMS staff discuss calls attended with FR (Yes/No);
5. FR receive written feedback on individual calls from the EMS (Yes/No);
6. FR participate in a debrief with EMS staff immediately after an OHCA (Yes/No).
7. Style of financial assistance from the EMS (either in the form of payment or equipment).
8. For the citizen-responders information was retrieved on whether emotional support was available (Yes/No).

**Supplementary 2, Description of the variation in implemented First-Responder systems on a national and regional level.**

On a national level, firefighter and citizen-responder are the most common type of First-Responders (FRs) reported, either dispatched alone or together. In Finland, Luxembourg, Norway, Poland, Portugal, and Slovenia, Romania, FR-systems with firefighters only are implemented nationally (or limited to specific regions) whereas the FR-systems in Hungary and Scotland include only citizen-responders. In Luxembourg and Slovenia, the firefighters act in a voluntary capacity. In Slovenia, the FR-system is primarily based on firefighters because of the extensive network of volunteer fire brigades across all villages. In Ireland, while citizen-responders make up the greater proportion of FRs, off-duty EMS personnel and medical doctors are also part of the FR-system. In contrast, Austria, Czech Republic, England, Italy (limited to some regions), the Netherlands, Sweden, and Switzerland have at least three of the following types of FRs in their FR-system: firefighter, citizen-responder, other dispatched responders.

As well as variation at a national level, variation within countries was also reported. For example, in Belgium FR-systems were limited to specific regions and one FR only: Brussels (police) and Antwerp (citizen-responders). In one region of Sweden, the FR-system consist of police (Halland) only whereas another Swedish region was limited to firefighters whereby these firefighters were only sent out if the dispatchers aiding tool calculates the response time to be in favour for the firefighters. Two other responding regions added a second responder to the firefighters. Hence their system includes both firefighters and citizen-responders. Multiple other regions added a second responder e.g. Copenhagen (Denmark) and Bucharest (Romania) dispatch a combination of firefighters and citizen-responders. Also, in the Romanian capital Bucharest, the FR-system includes both firefighters and citizen-responders, whereas in other parts of Romania, only firefighters act as FRs. In contrast, in the Netherlands, citizen-responders have for several years been part of the FR-system in all parts of the country.

In the Italian region of Emilia Romagna, the FR-system includes four different types of FRs, while no FR-system exist in the nearby province of Pavia.

In Germany, both EMS organized and non-EMS organized initiatives commonly exist. In multiple regions, EMS-organized initiatives are implemented dispatching several types of responders (commonly off-duty health care professionals and citizen-responders) by app. There are four different app systems in Germany. In several remote areas, community systems not connected to the EMS exist. However, the present study did not map these non-dispatched systems. In the Czech Republic, data presented in this study apply in particular to the Hradec Kralove region. Other regions in the Czech Republic dispatch citizen-responders, police officers, firefighters and off-duty EMS personnel, but in a different way. Moreover, although firefighters are part of the FR-system in the Czech Republic, they are only dispatched in extremely rare cases. Lastly, even though limited to only one FR type, variation across regions may occur. For instance, in Norway and Slovenia, firefighters are dispatched only. Also, all firefighters in Slovenia are volunteers. Therefore, density of firefighters acting as FRs varies across the entire country.

**Table S1, Characteristics of other responders**

| **Region** | **Description of responder** | **Method to activate the responder** | **Location determination** | **Training; *Frequency*** | **Registration** | **Feedback** | **Financial assistance** |
| --- | --- | --- | --- | --- | --- | --- | --- |
| **Austria**  (N=8.220.000) | Off-duty EMS personnel; Medical Students; Physicians | Dedicated communication system; SMS; Pager | GPS; Address | Yes trained for CPR including use of an AED; *Annually* | App; Dispatch centre | These first-responders receive a regular report of their activity; Designated EMS staff discuss calls attended with these first-responders | Yes, AED equipment and AED related disposables |
| **Czech Republic,**  (N=10.521646) | Off-duty EMS personnel | SMS; APP | GPS | Yes trained for CPR including use of an AED; *Annually* | APP: KISS SHARP (new name O2 SOS) at fr.zzskhk.cz | These first-responders participate in a debrief with EMS staff immediately after an OHCA | Yes, pocket mask and safety vest |
| **Germany,** County of Marburg-Biedenkopf  (N=241.598) | Off-duty EMS personnel, off-duty physicians, off-duty nurses, volunteer firefighters | SMS | Address | Yes trained for CPR including use of an AED; *Annually* | Registration by their organizations and the EMS medical director | - | First response system and equipment is completely funded by involved organizations |
| **Ireland**  (N=4.830.000) | Off-duty EMS personnel | SMS | Not determined | Yes trained for CPR including use of an AED; *Training is just for a few corporations and scattered throughout the country, not nationally organized* | Pre-hospital Emergency Care Council registry | Activity is described in the annual report; Participate in a debrief with EMS staff immediately after an OHCA | No |
| **Italy,** Emilia Romagna  (N=4.453.000) | Taxi drivers | APP | GPS | Yes trained for CPR including use of an AED; Bi-annually | App: smartphone Application and registration program created by Emilia Romagna Region Healthcare System | The responder manager sends an email to collect feedback by mail (but is not a routine) | Equipment and training is funded by involved organizations |
| **Switzerland - Ticino Canton**  (N=353.709) | Off-duty EMS personnel, Border Patrol, Military Police, Train Police | APP | Not determined | Yes trained for CPR including use of an AED; *Bi-annually* | APP: smartphone Application (MOMENTUM) and registration program created by us (fr.ticinocuore.ch) | These first-responders receive written feedback on individual calls from the EMS; These first-responders participate in a debrief with EMS staff immediately after an OHCA/Firefighters receive feedback based on AED data (HP CPR) from the operational and clinical manager. They are invited to the annual ceremony, where survivors meet first responders | AED and rescue kit donated free from Ticino Cure Foundation |
| **Switzerland, region of Fribourg**  (N=225.500) | Taxi drivers | APP | GPS | Yes trained for CPR including AED use; *Every two years* | Have an account in the app | Taxi drivers must complete a feedback sheet. Dependent of the answers, the foundation will contact the taxi driver. | No |

**Abbreviations: AED, Automatic External Defibrillator; ; APP, (mobile) application; CPR, Cardiopulmonary resuscitation; EMS, Emergency Medical Services; GPS, Global Positioning System; OHCA, out of hospital cardiac arrest; SMS, text message*

**Table S2, Countries without First Responders**

| **Country** | **Start to implement FR initiatives at the present moment** | **Specific information or reasons** |
| --- | --- | --- |
| Bosnia and Herzegovina | No | There is no system of FRs in Bosnia and Herzegovina. Police officers, firefighters or citizen-responders and other services do not participate in any medical rescue. Also, EMS may be less available outside major urban areas in the event of an OHCA. |
| Croatia | No | There is no system of FRs in Croatia. However, some small local initiatives exist. In these locations, firefighters and police officers are trained on a local basis but no national program has been implemented. Also, legislation about the use of AEDs by non-healthcare providers does not exist. At the present moment, Zagreb has nurses equipped with AEDs on motorbikes, but they are not systematically dispatched in the event of an OHCA. |
| Cyprus | Yes | There is no system of FRs in Cyprus. Cyprus has policies for OHCAs which are designed and implemented by the ministry of health of the Cyprus government.  The Cyprus Resuscitation Council has an advisory role at the moment and is responsible for the education of the community, and is responsible for the dissemination of the European Resuscitation Council initiatives. |
| Estonia | No | There is no system of FRs in Estonia. There are some local unmapped AEDs, but the location of these AEDs is not available to the dispatch centre. In public places, everyone can use AEDs. However, in some institutions, only designated persons can use it. |
| France | No | In France, as firefighters are part of the EMS, the implementation of other FRs may not be considered a priority. |
| Iceland | No | First responders are not dispatched in the event of an OHCA in Iceland at first instance. They are, however, dispatched in smaller rural areas on the islands, in smaller villages without EMS response. |
| Greece | Yes | There is no system of FRs in Greece. There are, however, many initiatives to train citizens, but a program to connect the EMS with citizens has not been established. There are AEDs in public places that can be used by anyone who is willing to do so. Also, in Greece, firefighters and police officers are trained on a local basis, but no national program has been implemented. Legislation about the use of AEDs by non-healthcare providers does not exist. |
| Malta | Yes | There is no formal FR system in Malta. However, dispatch centers do occasionally deploy community response to respond to OHCA cases when the response time is expected to be prolonged. The EMS response time is quite rapid given the small size of the country. A deal with the government to implement a community FR service is being negotiated. Apart from this, CPR training in the community is very active. |
| Serbia | No | The role of FRs acting in the community has not been defined officially. The concept of FRs is not yet considered a subject of interest and there is no legal background defined. |
| Spain | Yes | There are 17 public EMS centres. At least two of them are, at the present moment, implementing public volunteers for OHCA. |

**Abbreviations: AED, Automatic External Defibrillator; CPR, Cardiopulmonary resuscitation; EMS, Emergency Medical Services; FR, First-responder; OHCA, out of hospital cardiac arrest.*
